# Rediscovering the chick embryo as a model to study retinal development

**DOI:** 10.1186/1749-8104-7-22

**Published:** 2012-06-27

**Authors:** M Natalia Vergara, M Valeria Canto-Soler

**Affiliations:** 1 Wilmer Eye Institute, The Johns Hopkins University School of Medicine, Smith Building 3023, 400 N Broadway, Baltimore, MD 21287-9257, USA

**Keywords:** Chick, Retina, Development, RCAS, Morpholino, Gain of function, Loss of function, Transient transgenesis

## Abstract

The embryonic chick occupies a privileged place among animal models used in developmental studies. Its rapid development and accessibility for visualization and experimental manipulation are just some of the characteristics that have made it a vertebrate model of choice for more than two millennia. Until a few years ago, the inability to perform genetic manipulations constituted a major drawback of this system. However, the completion of the chicken genome project and the development of techniques to manipulate gene expression have allowed this classic animal model to enter the molecular age. Such techniques, combined with the embryological manipulations that this system is well known for, provide a unique toolkit to study the genetic basis of neural development. A major advantage of these approaches is that they permit targeted gene misexpression with extremely high spatiotemporal resolution and over a large range of developmental stages, allowing functional analysis at a level, speed and ease that is difficult to achieve in other systems. This article provides a general overview of the chick as a developmental model focusing more specifically on its application to the study of eye development. Special emphasis is given to the state of the art of the techniques that have made gene gain- and loss-of-function studies in this model a reality. In addition, we discuss some methodological considerations derived from our own experience that we believe will be beneficial to researchers working with this system.

## Review

### The chick embryo as a developmental model organism

#### A historical perspective

Avian embryos, and particularly the chick, have not only been instrumental to the field of developmental biology, but have also made significant contributions to the study of cell biology, virology, immunology, cancer biology and neuroscience. The discovery of NGF by Rita Levi-Montalcini, for which she was awarded the Nobel Prize in Physiology or Medicine in 1986 together with Stanley Cohen, is one of several Nobel Prize winning discoveries made using this model.

But the history of the chick as a developmental model organism started long before. Aristotle was the first to systematically study development by opening chicken eggs at different times and performing observations and dissections, which resulted in the production of the first great compendium of embryology in his book “*De Generatione Animalium*” (350 bc; reviewed by [1-5]). In the seventeenth century, with the aid of the recently invented microscope, Marcello Malpighi was able to perform a detailed description of several embryonic structures such as the somites, neural groove and blood vessels using the embryonic chick. And in the following 200 years, technical advances in histological sectioning and staining led to important contributions to the understanding of development and to the production of the first histological atlas by Mathias Duval in 1889 (reviewed by [1]). By the end of the nineteenth century, the realization by Wilhem Roux that experimental manipulations of embryos could provide important information marked another turning point in the history of embryology. Then followed stereoscopic time-lapse films, transplantation experiments, the use of chick-quail chimeras, electron microscopy and monoclonal antibodies, with each technological advance leading to further insights into the mechanisms of development ( [2] and references therein).

Many of the major concepts in developmental biology, such as those of induction, competence, plasticity and contact inhibition, are due to work done on the chick [6-8]. The first genes involved in left-right asymmetry and many transcription factors involved in dorso-ventral patterning were discovered using this system, and the same is true for the mechanisms that pattern the limb, the importance of somites in the segmentation of the peripheral nervous system and the mechanisms of brain segmentation in vertebrates ( [9-15]; reviewed by [2,5,16]; and others). Contributions of the chick model to other fields include the discoveries of the Rous sarcoma virus (RSV), the first cellular oncogene (c-src), reverse transcriptase, the mechanisms of RNA virus incorporation, and the division of T and B lymphocytes as functionally distinct populations, among many others [2,17-22].

#### Advantages and limitations

Some of the main characteristics of the chick embryo that have played a crucial role in its establishment as a research model include its significant similarity to the human embryo at the molecular, cellular and anatomical levels; its rapid development; its accessibility for visualization and experimental manipulation; and its comparatively large size and planar structure during early developmental stages.

When the egg is laid the chick embryo is at the blastula stage, and in only 2 to 3 days it will undergo gastrulation, neurulation and histogenesis, completing its entire development by the time of hatching at 21 days. This process has been documented in great detail thanks in part to the efforts of Hamburger and Hamilton, who provided a meticulous staging system for this animal [23]. Live optical imaging of the chick embryo can be accomplished through a small window in the egg shell, and in combination with a wide variety of cell marking techniques, it constitutes a powerful tool for tracking cell movements and fates in real time (for a practical guide on this matter see [24]). Another advantage of working with this model is the availability of an assortment of well-established experimental methods, including tissue ablation, rotation, auto- and allografting, implantation of beads coated with growth factors or small molecules, *ex ovo* culture of whole embryos, tissue explant culture and cell culture systems, among many others (reviewed by [25,26]; and others). Working with the chick offers the possibility of performing these manipulations at specific embryonic stages and allowing development to continue further by closing the window and re-incubating the egg, something that is more difficult to achieve in mammals.

What is more, the potential of this model has been further strengthened by the sequencing of the chicken genome. A high-quality draft assembly was released in 2006 [27], and NIH-supported efforts to bring this to a finished stage are underway. In addition, a large number of genomic resources are currently available to the research community, including sequence assemblies, linkage maps and a variety of databases for quantitative trait loci (QTL), SNPs and gene expression ontology, among others, which can be found at the "Chicken Genome Resources" database created and maintained by NCBI [28]. The sequencing of the chicken genome also revealed that this animal possesses roughly the same number of genes as humans, with a high level of sequence conservation, but in a much more compact diploid genome, characteristics that are very desirable for studies dealing with comparative genetics and the analysis of gene regulation and evolution (reviewed by [2,29,30]).

In addition, the economic and practical advantages of this system cannot be overlooked: the low cost of the eggs and their housing makes large-scale experiments more permissible than with other models, and more accessible to a wide range of laboratories. Moreover, eggs are available year round almost anywhere in the world, and they can be purchased in specific quantities, which facilitates the planning and scheduling of experiments. In connection to this, fertilized eggs can be stored in a cool place for a few days and then placed in the incubator at a particular time, allowing researchers to easily obtain embryos at the specific developmental stage that suits their needs.

Finally, focusing on the study of eye development in particular, the chick has once again been at the forefront of scientific research for a long time (in fact, it was the first animal model in which the features of this process were described, reviewed in [31]), contributing to much of our current knowledge on the topic. Adding to the attributes described above, chick eyes are particularly big compared to most other commonly studied animals, which constitutes an important advantage not only for surgical manipulations, but also for the collection of larger amounts of tissue for cell culture and molecular analyses. Moreover, embryonic chicks can regenerate their retinas at certain stages, making them also a good model for regenerative eye biology [32-38].

However, despite its numerous advantages, the chick has certain limitations, such as the difficulty to perform chemical mutagenesis screenings, which have proven to be very useful in other models such as Zebrafish and Drosophila. Yet probably the main challenge for working with the chick in the current age of molecular genetics has been the lack of a technology that allows for the efficient generation of genetically modified chicken lines. Nonetheless, important advances have been made in this regard in the past decade. Transgenic chickens have indeed been generated, and the strategies to obtain them have been greatly improved through the work of several laboratories (reviewed by [39-41]), including the generation of transgenic chickens expressing a tetracycline-inducible GFP gene [42]. Considering that chickens have a relatively fast generation time, that many offspring can be produced from one set of parents, and that it is easy to assess phenotypic differences, it is likely that several useful mutant chick lines will become available for biological research in the coming years.

On the other hand, transient transgenesis has been applied with great success in this model, leading to important progress in our understanding of developmental and molecular processes. Two technological advances developed over the past 20 years have been particularly significant in this regard: the use of the RCAS retroviral system for exogenous gene expression [43], and the devise of electroporation techniques that can be used to overexpress genes and reporter constructs, as well as to downregulate mRNA or protein levels by the use of dominant negative constructs, siRNA or morpholino antisense oligonucleotides [44].

Notoriously, transient transgenesis is perhaps one of the most important advantages offered by the chick as a developmental model system, since it allows for the relatively easy manipulation of the expression of one or more genes, sequentially or at the same time, in a tissue-specific manner, and with fine tuning of the developmental stage. This is something much more difficult to achieve in other transgenic or knockout models, because of the requirement for promoters to drive gene expression at precise times and in specific places, which are not always available, and for the cost and length of time required to generate double or triple mutants [1].

In the rest of this article, we will discuss some of the most useful technologies for transient transgenesis that are currently available for the analysis of the role of developmentally important genes in the embryonic chick, focusing particularly on their use for eye development studies. Instead of giving a broad yet superficial synopsis of all the possible techniques, we consider it more useful for the purpose of this review to concentrate on one major technique for each gain- and loss-of-function studies, discussing their advantages and limitations in more detail, and provide a brief overview of alternative approaches.

For gain-of-function studies we have chosen the RCAS retroviral system, which efficiently delivers genes into proliferating avian cells. For loss-of-function analyses we will discuss the morpholino technology, which has provided significant advances in our understanding of development in different mammalian and non-mammalian animal models. Finally, we will provide some examples of how these technologies can be used in combination with the more traditional embryological manipulations that are a strength of this system as well as with newer molecular and bioinformatics tools in order to create a unique experimental model for the study of development.

### The chick embryo as a model to study eye development

#### Brief description of eye development

Vertebrate eye development is a complex and dynamic process that results from the combinatorial action of many factors and cellular interactions among different tissues in order to generate highly organized and specialized structures. We will present here a brief overview of the mechanisms involved in this process in order to provide a framework for the following discussion. For a more thorough description, the reader is referred to some excellent reviews that have been published on the topic [45-50].

**Figure 1:**
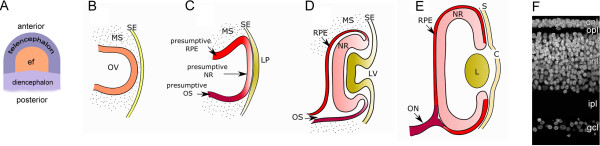
**Schematic representation of vertebrate eye development. **(**A**) Specification of the eye field within the anterior neural plate. (**B**) Formation of the optic vesicle. (**C**) Specification of the RPE, neural retina and optic stalk domains within the optic vesicle and formation of the lens placode from the surface ectoderm. (**D**) Formation of the optic cup and the lens vesicle. (**E**) Mature optic cup and lens. (**F**) Organization of the mature retina. Abbreviations: C: Cornea; ef: eye field; gcl: ganglion cell layer; inl: inner nuclear layer; ipl: inner plexiform layer; L: lens; LP: lens placode; LV: lens vesicle; MS: mesenchyme; NR: neural retina; ON: optic nerve; onl: outer nuclear layer; opl: optic plexiform layer; OS: optic stalk; OV: optic vesicle; RPE: retinal pigment epithelium; S: sclera; SE: surface ectoderm. Modified with permission from Adler and Canto-Soler, 2007.

The initial steps in eye development take place during late gastrulation, when a region of the anterior neural plate becomes specified as the "eye field" under the induction of the prechordal mesoderm (Figure 1A). The eye field is then divided into two separate lateral domains by the action of signaling molecules secreted from the midline prechordal region. The first morphological indication of eye development is the evagination of the optic vesicles (OV) from the eye field-derived lateral domains (Figure 1B). Each OV then expands through the mesenchyme and makes contact with the surface ectoderm, at which point a cellular and molecular cross-communication is established between these tissues, resulting in complex structural changes on both parts (Figure 1C-E). The ectoderm thickens to form the lens placode, which later invaginates, giving rise to the lens vesicle, whereas the surface ectoderm progresses towards the formation of the cornea. During the course of development, the cells that constitute the lens vesicle will become more specialized and differentiate to form the lens epithelium in the anterior region, and the lens fibers in the posterior part, resulting in functional lenses. Invagination of the lens placode occurs simultaneously with the invagination of the optic vesicle to form the optic cup (OC). Upon invagination, the OC becomes a bilayered structure connected to the dinencephalon through the optic stalk. The internal layer of the OC will give rise to the neural retina, the light-sensing and -processing tissue of the eye, while the external layer will become the retinal pigmented epithelium (RPE); the hinge region in turn will form the iris and ciliary body.

The retina itself is subject to a progressive process of differentiation that starts with the specification of the "neural retina domain" within the optic vesicle neuroepithelium, composed of proliferating, undifferentiated retinal progenitor cells (Figure 1C). Following optic cup formation and throughout the course of development, these progenitors differentiate in a sequential yet overlapping manner into seven different types of neurons and glia, giving rise to a laminated structure with the cell bodies organized into three nuclear layers separated by the plexiform layers created by their synaptic projections (Figure 1F). Evidently, achieving this level of complexity requires the fine regulation of multiple cellular processes including survival, proliferation, cell fate specification, migration, axonal pathfinding and synapse formation. Such feats can only be accomplished by the orchestrated action of multiple and overlapping intercellular signaling molecules, and cell-intrinsic mechanisms involving a delicate interplay of transcription factors and epigenetic modifications. In the following subsections, we will discuss some of the tools that are available to researchers for the analysis of these complex regulatory mechanisms.

### In vivo gain of function by retroviral gene transfer

#### The RCAS system. Characteristics and experimental considerations

The term RCAS stands for Replication-Competent Avian sarcoma-leukosis virus (ASLV) long terminal repeat (LTR) with a Splice acceptor vector. These laboratory-derived retroviral vectors are capable of infecting and delivering exogenous genes into avian cells, and have been extremely valuable tools in chick developmental studies since their creation in the late 1980s.

Retroviruses are composed of a single-stranded RNA genome encased in an enveloped capside that also contains the enzymes reverse transcriptase and integrase. Infection occurs when the glycoproteins in the envelope are recognized by specific receptors on the cell surface. This event triggers the fusion of the viral membrane with the cell membrane and the release of the viral core into the cytoplasm of the host. Once there, the viral RNA is uncoated and reverse transcribed, producing a linear double-stranded DNA that contains LTRs in both the 5' and 3' ends, and the genes *gag* (encoding structural proteins for the matrix and the capsid), *pol* (encoding reverse transcriptase and integrase) and *env* (encoding the envelope glycoproteins). This DNA can then enter the nucleus during M phase and integrate into the host genome (at which point it is called a "provirus"), from where it can be transcribed. Once viral proteins are translated they are transported to the cell surface together with a portion of the transcripts, and assembled into new infectious viral particles to be released from the host cell [43,51].

In nature, retroviruses can sometimes acquire oncogenes from their hosts. Such was the case of the Rous sarcoma virus, which co-opted the cellular gene *src* while still retaining all the genes necessary for the viral replication cycle. RCAS vectors are derived from the SR-A strain of this virus, but the oncogene *v-src* has been eliminated and replaced with a *Cla*I restriction site, so that an exogenous gene can be inserted in its place [43,52,53]. These vectors replicate efficiently in avian cells, yet they are constitutively replication-defective in mammalian cells [43], making them quite safe for laboratory use. Moreover, the fact that they allow the stable integration of the exogenous gene into the host genome means that the transgene is continuously expressed and there is no dilution with cell division; on the contrary, infection continues spreading throughout development. These characteristics are particularly desirable when long-term expression of a transgene is required.

RCAS, RSV and ALV (avian leukosis virus, from which RSV originated) are all members of the ASLV family. This family is divided into ten subgroups (designated by the letters A-J) according to the type of glycoprotein displayed on the viral envelope, which in turn determines receptor specificity [54]. When a cell is infected, expression of the viral envelope glycoprotein will block the receptors on the cell surface, preventing superinfection by another virus from the same subgroup, a mechanism known as receptor interference. This phenomenon needs to be considered when designing an experiment that requires the use of more than one vector carrying different inserted genes. Moreover, it should also be remembered that not all chick strains possess functional receptors for all the different ASLV envelope glycoproteins [55-57]. For these reasons, RCAS vectors expressing different envelope genes (A-E) have been designed.

A further subdivision of this family is into exogenous and endogenous viruses. Most of the subgroups are composed of "exogenous" viruses, implying that infection can be transmitted horizontally from individual to individual or vertically to progeny. The exception is the members of subgroup E, which are "endogenous" because their genome has been integrated into the host germ line, and therefore they are transmitted in a Mendelian fashion (reviewed by [43,58,59]). These endogenous proviruses are encoded in the endogenous provirus *(ev) loci*. The fact that most chicken strains, including those commonly used in developmental biology studies, contain *ev loci* deserves special consideration for various reasons including the tendency of retroviruses to recombine with other closely related retroviruses, which can result in unwanted recombination between the RCAS vector and the endogenous provirus [59,60]. Moreover, we have observed that certain viral proteins commonly used to identify experimentally infected cells can sometimes be expressed from these *ev loci*, which complicates the analysis of results [59].

Different types of RCAS vectors have been created to allow greater flexibility in their applications. When a gene is inserted in an RCAS vector, its expression is driven by the viral LTR promoter. The level of expression is therefore affected by the enhancer in the LTR, but also through a mechanism that is not completely understood, by the sequence of the *pol* gene [43]. Different modifications have been made to the original vector in order to modulate the level of expression of the inserted gene: replacement of the LTR region for that of the endogenous retrovirus RAV-O resulted in the creation of RCOS vectors with low enhancer activity, whereas substitution of the RCAS *pol* gene with that of the Bryan high-titer strain of RSV produced the RCAS-BP vectors (RCAS Bryan Polymerase), which increase the titer and transgene expression by 5–10 fold over standard RCAS [61-63]. In addition, for applications requiring expression of the inserted gene under the control of a non-viral promoter, the RCAN vectors (Replication-Competent ASLV LTR with No splice acceptor) are available [43]. Newer versions of these vectors include multiple cloning sites for gene insertion, and some are compatible with the Gateway system to facilitate cloning [64]. What is more, a tetracycline-inducible element has been inserted into RCAN-BP vectors to allow for inducible expression of an inserted gene ( [65]; in [42] this type of system was used to generate transgenic chickens). Finally, replication-defective vectors have been made available for specific applications, such as lineage tracing and fate mapping [66-68].

The RCAS system does, nonetheless, have some drawbacks. Among them is the fact that these vectors do not infect non-dividing cells efficiently*,* which is an important point to consider in experimental design. In the chick embryo model, most cells are actively proliferating at early stages and can therefore be infected with these vectors; thus, as development progresses and different cell populations start exiting the cell cycle, those that had already been infected will continue to express the transgene. However, if infection is attempted at later stages, it will selectively affect those populations that are still actively dividing. Moreover, the time lag between virus administration and transgene expression needs to be carefully considered: once the virus has been administered, only a subpopulation of cells will be effectively infected; the extent of this initial infection will depend on several characteristics of the system, such as the viral titer and volume injected and the number of proliferating cells. After that, the rate of production and release of viral particles by infected cells will depend on other factors, such as the length of the cell cycle, site of proviral integration and strength of the promoter/enhancer [43]. Therefore, the choice of the RCAS technology may be inadequate for studies in which phenotypical changes need to be assessed shortly after viral injection.

Another limitation of this system is that the insert size is restricted to a maximum of 2.4 kb. This makes it difficult to insert large constructs, such as very large genes (which may not occur frequently since most genes fit within the allowed size range), or constructs containing two genes, such as fusion proteins or bicistronic systems.

Despite these limitations, the RCAS system has been, and still is, a fundamental tool in developmental studies using the chick model. In the field of eye development, much of our current knowledge can be attributed to work done with this system, as for example some of the mechanisms behind the patterning of eye structures [69,70], the distribution of axon guidance molecules [71], as well as the role of important signaling pathways in optic cup development [72-75] and in retina regeneration [35-38].

The RCAS website [76], hosted by the National Cancer Institute and maintained by Stephen Hughes (one of the original developers of the RCAS system) and his group, is a very useful resource for those researchers interested in applying this technology. Many RCAS constructs, including gateway-compatible destination vectors, are available from Addgene [77].

#### Screening and selection of appropriate egg lines

The implementation of the RCAS virus technology for developmental studies requires the use of certified "Specific Pathogen-Free" (SPF) eggs. These are fertilized chicken eggs derived from controlled parent flocks that are certified to be free of antigens belonging to several pathogens including the ASLV family. They are produced and maintained following specific biosafety standards, since they are used, among other things, in the production and control of vaccines for humans and animals.

However, we have recently demonstrated that not all certified SPF chicken lines meet the minimal requirements to ensure proper interpretation of research results [59]. In fact, we tested SPF certified White Leghorn eggs (the strain most commonly used in developmental biology) from three different commercial breeders in the US, and found that three of the four different flocks tested were positive for the ASLV viral proteins p19 and p27, as assessed by immunohistochemistry. This is particularly important considering that, according to a survey of the literature from the past 10 years, expression of these proteins has been used to assess RCAS vector infection in cells or tissues in the majority of research articles using this system, under the assumption that they would not be expressed in wild type SPF quality embryos. Our results suggest that conclusions based on the presence of these proteins to pinpoint transfected cells need to be taken with caution and that care should be taken in future studies to avoid this kind of potential conflict.

It is important to mention that in our study, the extent and pattern of viral protein expression, as well as the percentage of embryos displaying it, varied not only among different flocks, but also among embryos within the same flock. However, even those embryos that expressed ASLV proteins were unable to produce either exogenous or endogenous viral particles, indicating that the expression of viral proteins in tissues can sometimes occur independently from virion production. In fact, our genetic screening demonstrated that almost all the embryos analyzed (24/25) from the four different flocks contained multiple *ev loci* regions and that there was great heterogeneity in *ev loci* composition even among embryos of the same flock [59].

As mentioned before, the concern goes beyond ensuring the appropriate identification of RCAS transfected cells. At least 23 different ASLV *ev loci* have been identified in the genome of White Leghorn chickens [78]. These include both defective and non-defective retroviral inserts whose expression depends on several factors, including the completeness of the proviral genome, the site of integration, the genetic background and epigenetic modifications, such as DNA methylation ( [79]; reviewed in [59]). In that context, treatments that alter epigenetic states can induce the generation of viral particles from previously silent *ev loci *[79]. In addition, the genetic heterogeneity of the chicken lines increases the chances for stochastic genomic recombination, which can lead to both de-novo production of infectious virions from previously defective *ev loci* and undesired recombination events for the experimental RCAS vectors. How these matters can affect research results depends on the context, but they should be taken into account in the experimental design.

**Figure 2:**
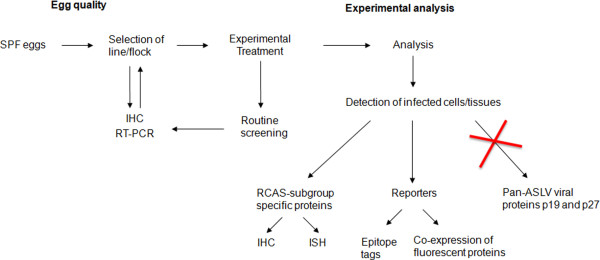
**Recommended guidelines to ensure egg quality for research purposes. **SPF eggs should be screened for the absence of ASLV viral particles and endogenous viral protein expression. Once an appropriate source of eggs is selected, screening should be performed routinely to ensure quality maintenance. After experimental treatment, infection can be assessed by immunohistochemistry (IHC) or in situ hybridization (ISH) designed to detect a specific RCAS subgroup, or by the use of reporter constructs. Originally published in McNally et al., 2010. Reproduced with permission.

Therefore, the selection of SPF lines devoid of endogenous viral protein expression is critical to ensure egg quality for research purposes. In order to standardize and facilitate this process, we have developed a series of practical tools and guidelines (see Figure 2).

(1) Characterization and selection of chicken strains

A source (line/flock) of research quality SPF eggs should be selected by screening for lack, or minimal detection if otherwise not possible, of ASLV viral particles and endogenous viral protein expression in tissues. We have developed a simple RT-PCR protocol for virion detection that can be completed in just a few hours using amniotic fluid samples ( [59]; Additional file 1). As for endogenous protein detection, we recommend the use of immunohistochemistry, yet taking into account that the heterogeneity in expression may lead to false negatives if not enough embryos/sections per embryo are analyzed.

(2) Routine screening of SPF lines/flocks in use

It is important to keep in mind that chicken flocks have limited productivity (30–40 weeks [80]; B&E Eggs, personal communication), which means that even when receiving embryos of a given line and breeder, periodic variations in the flock source are inevitable. Therefore, once a line is selected, routine screening needs to be performed to ensure that those conditions are maintained. If at any given point de-novo ASLV viral particle production and/or viral protein expression in tissues is detected, a new line or flock should be tested and selected for further use.

#### Technical considerations regarding vector design, preparation and delivery into the developing eye

The first step for the success of the experimental strategy is to ensure an appropriate vector design. Stephen Hughes, in his thorough review of the RCAS system [43], suggests a number of guidelines to prevent potential problems. These include: avoid inserting sequences that could interfere with the expression of the viral genome (such as stop codons or polyadenylation); avoid the insertion of repeat elements, since the nature of the reverse transcription process would lead to deletion of the region between highly homologous sequences; be cautious about inserting sequences whose protein products could be toxic to the host cell; and finally, observe the maximum size limit for the insert. Violation of these principles could lead to loss of insert and selective growth of empty vectors, since viruses with smaller genomes replicate faster.

The next step is the propagation of the vector in avian cells. This used to be routinely done in primary chick embryonic fibroblast (CEF) cultures, derived from the EV-0 strain of White Leghorn chickens. The important characteristic of this strain, maintained by the US Department of Agriculture, is that it is devoid of endogenous proviruses of the ASLV family, a feature that is necessary in order to avoid undesired recombination events. Only two other chicken lines have been developed with this characteristic: the 0-TVB*S1, derived from EV-0, and the Canadian WG line [81]. Currently, most developmental biology laboratories working with the RCAS system can take advantage of the DF-1 cell line for vector propagation [82]. This chicken fibroblast cell line was derived from EV-0 animals and is available from the American Type Culture Collection (ATCC). DF-1 cells are easy to maintain and can be transfected with RCAS plasmids using standard protocols. In our laboratory we routinely use lipid-based transfection for this purpose. Once the virus starts replicating, infection spreads efficiently to all the cells in the culture. The supernatant containing viral particles can then be collected, rid of cellular debris by centrifugation and, if desired, concentrated (for a detailed protocol, see Additional file 2; and [59,83,84]). Note that even though the cells can be infected with a previously produced virus stock, this practice is not recommended. This is because the process of reverse transcription is not always faithful and there is a small but real chance of losing the inserted gene, resulting in the amplification of empty virus. The likelihood of such outcome increases with repeated rounds of infection, and therefore it is more prudent to generate fresh virus stocks by transfecting cells with the plasmid construct encoding the virus. Alternatively, it is also acceptable to continue passaging the infected cells, since at that point the provirus is stably integrated in their genome [43].

Finally, to achieve efficient infection in vivo, a viral titer (number of infective particles per unit of volume) of 10^6^-10^8^ is recommended. The viral titer can be assessed by exposing DF-1 cells to serial dilutions of the viral stock and assessing infection (Additional file 3 and [84]).

**Figure 3:**
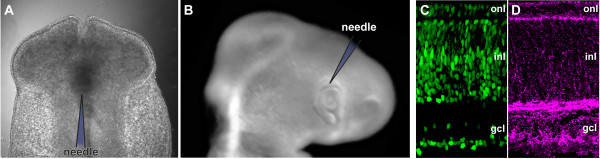
**Gain of function by retroviral gene expression. **Viral solution is injected into the anterior neural tube of ED1.5 chicken embryos (**A**) or in the vitreal cavity of ED3 chicken embryos (**B**). (**C-D**) Transversal section of the retina of an ED12 embryo that was injected with RCAS construct expressing GFP on ED3. (**C**) Shows GFP expression (green) throughout the layers of the retina; (**D**) detection of viral infection by an antibody against RCAS subgroup A envelop protein (magenta). Abbreviations: gcl: ganglion cell layer; inl: inner nuclear layer; onl: outer nuclear layer.

For studies on eye development, and particularly retina development, RCAS viruses are usually injected in the anterior chick neural tube between stages 9 and 12 (embryonic day [ED] 1.5), while the optic vesicles are developing (Figure 1B-C) or at stages 17-18 (ED3; Figure 1D) when the eye cup is already formed by injecting either in the vitreal cavity or in the subretinal space, depending on the purpose. A simple protocol can be followed to perform these injections:

1. The eggs are incubated on their side until the desired stage, at which point a window is cut on the egg shell to allow access to the embryo. A detailed video of the protocol most commonly used has been published in the Journal of Visualized Experiments [85]. We recommend not discarding the cut egg shell lid, since in our experience, survival of the embryos is highly increased when the eggs are closed again using this lid (step 4).

2. The embryo can be observed through the window using a dissecting scope. We find that blue light illumination greatly enhances contrast, which improves visibility especially at very early stages, eliminating the need for injecting ink or other contrast solutions that can be toxic for the embryo. This can be achieved by attaching a blue dichroic filter to a regular fiber optic lamp as described in [86]. Embryos are staged according to H&H [23], and once the neural tube or the eye is located, injection can be performed.

3. We inject a viral stock solution with a titer of 10^6^-10^7^ infectious particles/ml (fast green can be added to a concentration of 0.005% to improve visualization), using glass capillary needles attached to a microinjector with a foot pedal (PLI-100, Harvard Apparatus, Holliston, MA, USA). Needles are made by pulling and beveling a borosilicate glass capillary (TW100-4, World Precision Instruments, Inc., Sarasota, FL, USA) to a pore diameter of 12 μm. Beveling is particularly important when performing injections inside the eye (at ED3 or later) in order to avoid unnecessary damage, since the tissues are harder at this point. The microinjector is set to deliver 0.25 μl of solution in 20 injection pulses. For neural tube injections, the needle is oriented parallel to the neural tube, and the solution delivered directly into the ventricle, whereas for intravitreal injections, the needle is introduced from the nasal side of the eye at a 45° angle (Figure 3A-B).

4. After injection the lid is closed and secured with tape, and the eggs are placed on their side in the incubator until the desired collection time.

Even though this methodology is intended for eye infection, a similar approach can be applied to injections in other parts of the central nervous system, as well as other tissues and organs.

#### Analysis: do's and don'ts

Since not all the cells in a tissue may have been infected with the virus and thus express the transgene, it is important to accurately identify the infected cells in order to properly analyze the experimental results. This has been traditionally accomplished by immunohistochemistry to detect the pan-ASLV envelope proteins p19 and p27, but since as we already discussed SPF embryos are capable of expressing the endogenous form of these proteins, this approach is no longer recommended because it can lead to false positives. An alternative strategy, founded on the premise that SPF eggs are indeed free of exogenous viral particles and the fact that the most commonly used RCAS vectors are derived from exogenous subgroups, is to base the identification on the detection of subgroup-specific proteins [59]. We are aware of the commercial availability of antibodies against RSV subgroup A and B glycoproteins (Charles River), and we currently apply this method in our laboratory with great success (Figure 3C-D). In those cases for which antibodies are not available, in-situ hybridization subgroup-specific probes can be easily designed, since the sequences for the ASLV subgroup genes have been well characterized.

Another valid approach, when the insert size allows, is to express the gene of interest fused to an epitope tag (such as influenza hemaglutinin, HA) or to co-express a fluorescent reporter (such as GFP or RFP) from the same transcript [59]. Figure 2 summarizes these recommendations.

Finally, even though integration into the host genome is a desirable characteristic for many experimental purposes, it should be noted that this can potentially result in the disruption of endogenous genes or regulatory sequences. The likelihood of such events, however, is very low, because: (1) integration of this type of viruses is mostly random, so a large proportion of the viruses will be integrated in intergenic regions as opposed to other retroviruses that tend to integrate in or near genes [87]; (2) even if viral integration disrupts a gene, this is often times inconsequential in diploid organisms such as the chick [43]. However, the rare possibility that the insertion site of the viral genome could lead to an unspecific phenotype should be taken into account and compensated for by analyzing reasonably large sample groups.

#### Alternatives for gain-of-function studies

It is evident that the RCAS retroviral system has been a fundamental tool for developmental studies in the chick embryo. However, other viral vector systems have been developed that present certain advantages over RCAS, but that are not devoid of their own limitations. The successful choice of exogenous gene delivery method depends on the careful consideration of the characteristics of each system as well as on the biological process under investigation.

Lentiviral vectors are retroviruses that, like RCAS, are able to integrate into the host genome, providing stable, long-term expression of the transgene, yet they are different in their ability to infect both dividing and non-dividing cells efficiently, so that they can be used to target terminally differentiated cells. These viruses have the capacity to infect human cells and therefore they are designed to be replication defective, requiring cotransfection of lentiviral packaging and expression vectors in a helper cell line in order to generate infective particles. This entails a longer and more complicated process for the generation of vectors for experimental use, and more stringent biosafety guidelines need to be followed for their manipulation in order to avoid potential generation of replication-competent viral particles. In addition, infection with replication defective viral particles implies that infection will not continue spreading horizontally to other cells during development but only vertically to progeny. Lentiviral vectors have been used to generate germline transgenic chickens [88-91], and several types of self-inactivating and bicistronic lentiviral constructs for studies in the chick have been developed [92-97].

Another alternative for this type of studies is the use of adenoviral vectors. These are DNA viruses also capable of infecting both non-proliferating and cycling cells. Moreover, they can be produced at very high titers, and they allow for relatively large insert size. Their main limitation resides in their inability to integrate their DNA into the host genome, which implies transient transgene expression, making them good candidates only for short-term studies.

Finally, a different and widely used approach to gain-of-function studies in the chick is the electroporation of plasmid constructs. Electroporation involves the use of an electric current to transiently open pores on the cell membrane, allowing the uptake of plasmid DNA into the cytoplasm. This is a very powerful technique that provides numerous advantages for developmental studies [44,98]. In particular, electroporation allows better target-tissue control because of its directionality, since the plasmid DNA exposed to an electric current will migrate towards the positive electrode, so that strategic choice of injection site and placement of the electrodes can be combined to achieve an efficient and relatively localized transfection of a region of interest [99]. Due to the nature of this mechanism, electroporation is not limited to dividing cells, and concomitant introduction of two or more plasmids is easily achieved without the problem of receptor interference that viral systems have. In addition, plasmids allow a wide range of sizes for their inserts, which provides greater flexibility, especially for cases in which more than one gene needs to be expressed from the same construct. What is more, this technique can be used to deliver plasmids that carry the gene of interest under a constitutive, cell-type specific or inducible promoter/enhancer, or plasmids designed to study promoters and enhancers by driving the expression of reporter genes ( [100-109]; and others). For example, Hilgers et al. combined electroporation with the tetracycline-dependent inducible Tet-Off system to study the effects of the 3'UTR in mRNA stability in the embryonic chick [110], whereas Watanabe et al. used electroporation of Tet-On and Tet-Off constructs to elucidate previously unknown roles of certain genes during chick somitogenesis [111].

However, since the plasmid DNA is not incorporated in the genome, expression of the transgene is transient and subjected to a dilution effect on the plasmid content as cells continue to divide. In addition, some commonly used promoters, such as CMV, become silenced after time. Other important limitations include the inability to control the amount of plasmid incorporated in each cell, which allows for a certain variability in transgene expression levels from cell to cell, and the difficulty of electroporating older embryos, since as the chick develops it tends to turn inwards, complicating the correct placement of the electrodes and making some tissues inaccessible for manipulation. Some of these limitations could be overcome by using plasmid constructs coupled to a transposon system [112] or by electroporation of plasmid vectors encoding RCAS viruses. It is important to notice, however, that contrary to electroporation of non-viral DNA plasmids, with most RCAS viruses this approach does not guarantee a spatially restricted expression of the transgene, since the RCAS virions will continue to replicate and infect other cells. Spatially restricted transgene expression can be achieved by electroporation of replication-defective proviral vectors or replication-competent proviral vectors into embryos insensitive to the corresponding viral subgroup [99].

For detailed protocols and tips on the electroporation of plasmid constructs, the reader is referred to some excellent articles on the topic ( [44,98,99,103,113-115]; and others).

### In vivo loss-of-function using morpholino antisense oligonucleotides

#### Morpholino technology

Morpholinos (MO) are synthetic nucleic acid analogs in which the sugar moiety has been replaced by a morpholine ring [116]. They normally consist of 25 subunits linked together, and unlike nucleic acids, the morpholino phosphorodiamidate backbone is uncharged. Conveniently, the ends of MO oligonucleotides are named 3' and 5' by analogy to nucleic acids, even though following IUPAC rules the numbers of the end carbons would be different [117].

The use of MOs in loss-of-function strategies is based on their ability to bind to specific, complementary RNA sequences, but they differ from other antisense reagents in that they do not recruit RNAseH or the RISC complex, but rather pose a steric hindrance on the processing or translation of their target RNA. MOs were originally designed as potential therapeutic reagents, and thus they display very good water solubility and low toxicity [116]. Moreover, these polymers are very stable since they are not subject to degradation by nucleases or proteases (reviewed by [118]; and references therein).

Morpholinos have been used in developmental biology research, and particularly in embryonic chick studies, with great success [86,119-121]. The most commonly used method to deliver MOs in live chicks *in ovo* is through electroporation. In addition to the advantages of this technique that have already been discussed, this strategy allows for a rapid knockdown of protein levels in the targeted tissue. What is more, as in the case of plasmids, multiple MOs targeting the same or different RNAs can be delivered at the same time, providing great flexibility for combinatorial knockdown experiments.

It should be noted that, upon entering the cells, MOs start effecting their inhibitory action immediately. However, they will not affect pre-existing proteins, and thus the time required to observe phenotypic consequences might be delayed depending on the turnover rate of the specific protein. In addition, the intracellular concentration of MOs will be diluted with cell division, decreasing their effect and making them more efficient for short-term experiments.

#### Designing and working with morpholinos

Different strategies can be devised for loss-of-function experiments using morpholinos. They are most commonly designed as either mRNA translation-blockers or splice-blockers, although they can also be used to interfere, for example, with microRNA function either by directly binding to them (thus preventing them from binding their targets) or by competing for the mRNA sequences they would normally bind.

Translation-blocking MOs should be designed to target the region between the 5'-UTR and the first 25 coding bases of a specific mRNA. Once bound, they can stop the progression of the initiation complex toward the start codon, preventing the assembly of the ribosome and halting the process of protein translation. Splice-blocking MOs on the other hand are intended to interfere with the proper splicing of pre-mRNA, and thus they should be designed to target intron-exon junctions (complementing primarily the intronic portion), preventing snRNP binding and subsequent spliceosome assembly. With the goal of eliminating protein activity, splice-blocking MOs can be designed in a way that causes the excision of an exon that is critical for the protein's function, or the inclusion of an intron that contains a stop codon or that will result in a shift in the reading frame. Such strategies require good knowledge of the protein's structure and function, and reliable intronic and intron-exon junction sequences [117].

Further considerations are important in MO design: First of all, it is essential to perform a BLAST or similar homology search, to ensure that the selected target sequence is not homologous to sequences in other mRNAs, which would give rise to undesired off-target effects. In addition, re-sequencing of the target region in the mRNA is advised to ensure proper complementarity, since sometimes errors can be found in the sequences deposited in public databases, particularly when those sequences are located in the 5'UTR. Other parameters to be considered include: G content, which should be limited to a maximum of 36% to avoid loss of solubility; percentage of GC, which should range between 40-60% to ensure good affinity without favoring non-specific binding; and self complementarity, which could cause loop formation or MO dimerization [117,122-124].

Modifications can also be made to MOs, usually in the form of additions to the 3' end, to facilitate their visualization in a tissue or a cell. These include the incorporation of a fluorophore, such as carboxifluorescein (emission wavelength 525 nm) or lissamine (sulforhodamine B, emission wavelength 593 nm), a biotin group or a primary amine that would permit the linking of other compounds.

Morpholinos are sold as a lyophilized powder that can be resuspended in sterile water. It is important that the water used for resuspension does not contain active DEPC, since it could react with the adenines in the MO and compromise its efficacy. Isotonic buffers can also be used, but they might decrease solubility and make reconcentration more difficult. A 1 mM concentration is suggested for a stock solution, which can be stored at room temperature. Storing MOs at lower temperatures is possible, but it can lead to a loss of activity due to precipitation. To avoid this problem, frozen or chilled aliquots can be reheated at 65°C for 10 min to re-dissolve possible precipitates.

Morpholinos for research use are commercialized exclusively by Gene Tools LLC, which also provides a free oligo design service. Protocols for handling, storage, concentration determination and delivery, as well as other useful resources and information can be found at the Gene Tools website [125].

**Figure 4:**
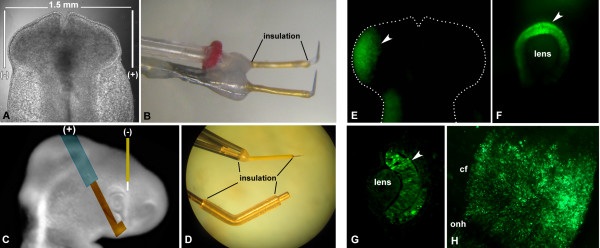
**Loss-of-function by morpholino antisense oligonucleotides. **(**A-B**) Setting and electrodes utilized for electroporation at ED1.5. (**C-D**) Setting and electrodes utilized for electroporation at ED3-4. (**E-G**) Images from embryos electroporated with fluorescein-labeled morpholinos at ED1.5. (**E**) Dorsal view of a whole mounted embryo fixed immediately after electroporation; (**F**) Side view of a whole-mounted embryo fixed 24 h after electroporation; (**G**) transversal section of the eye of an embryo fixed 24 h after electroporation. Arrows indicate morpholino incorporation (green) into retinal progenitor cells. (H) Flat mount of a retina electroporated with morpholino at ED4 and collected 24 h later. Abbreviations: cf: choroid fissure; onh: optic nerve head.

Electroporation is an efficient way to deliver MOs into various developing chick tissues. For eye development studies, this procedure is usually done at ED1.5 or ED3-4. The concentration of the MO working solution needs to be determined experimentally as it's efficiency will vary depending on several factors, including the abundance of the target mRNA. Also, the addition of fast green to this solution is not recommended since it has been reported to inhibit the uptake of MOs [120]; however, addition of a contrast dye is not necessary when using MOs carrying a fluorescent tag, as they are easily visible, especially under blue light. The protocol for injecting MOs in the neural tube (ED1.5) or the eye (ED3-4) is similar to that described for RCAS virus injections (see above). Once the working solution is injected in the desired location, an electric current transiently permeabilizes cell membranes, and the MOs are easily incorporated in the cells due to their small size. The following is a brief description of the electroporation procedure:

1. An electroporator with a foot pedal attachment is connected to the appropriate set of electrodes (we use an ECM 830 electroporator, BTX, Holliston, MA, USA).

2. Before electroporation, a small drop of HBSS (Hanks' Balanced Salt Solution) is applied on top of the embryo to prevent overheating and sticking of the electrodes to the tissues.

3. For electroporations on ED2, two thin platinum iridium electrodes (catalogue no. UE-PMEEVNNNND, FHC, Bowdoin, ME, USA) with a 1.5-2 mm gap distance between them are placed parallel to the embryo, on either side of its head (Figure 4A-B). It is important to avoid touching the optic vesicles with the electrodes, since that could cause damage to the tissues, preventing them from developing normally. For electroporations on ED3-4, a thin platinum iridium electrode connected to the cathode (−) is inserted perpendicular to the plane of the embryo, in a region of the head adjacent to the dorso-nasal portion of the eye, whereas a thicker gold-tipped electrode (catalogue no. 45-0115, BTX, Holliston, MA, USA) connected to the anode (+) is placed near the ventro-temporal region of the eye (Figure 4C-D). In our experience, placing the negative electrode closer to the heart decreases survival by interfering with heart function. Notice that all but the tip of the electrodes should be insulated for the system to perform efficiently.

4. In either case, three square pulses of 18 V, 50 ms in length, with a 950 ms interval, are delivered. Bubbles are generally observed, especially around the positive electrode, indicating that the electric current passage was successful.

5. Immediately following electroporation, another drop of HBSS is applied to the embryo to cool it down, prevent drying and remove air bubbles.

6. The egg is closed as explained before and returned to the incubator until the desired collection time.

### The use of appropriate controls

When working with MOs in laboratory research, every effort should be made to minimize the chances of causing toxic or off-target effects (non-specific binding to similar RNA sequences). In zebrafish, off-target effects have been reported to occur in as many as 15-20% of the cases (reviewed by [118,123,126]). Similar off-target effects have not been reported in the chick so far, and it is possible that the higher temperature at which eggs are incubated helps reduce the incidence of such events, but this problem should still be addressed as thoroughly as possible.

Determining the right dose is a good starting point, but it can be difficult in this system, as it is impossible to determine the amount of MO taken up by the cells when doing electroporation in highly multicellular tissues, and there can be considerable variability among embryos in this matter. However, taking into account that a high MO concentration can increase the odds of generating toxic and off-target effects, it is advisable to test a range of concentrations using the same injection and electroporation parameters (0.1, 0.5 and 1 mM could be used as a starting point), and then continue to work with the lowest effective dose.

Additionally, the selection of proper positive and negative controls is a crucial part of the experimental design. Controls should be performed in parallel and under the same conditions as the specific experimental MO. A strongly recommended (and economic) negative control is the *standard control oligo* sold by Gene Tools. This MO was developed to target a beta-globin intron mutation that causes beta-thalassemia in humans, and therefore should not have any specific effect in chick embryos. Most importantly, it has been thoroughly tested by many laboratories without showing toxic, teratogenic or non-specific activity.

Another good negative control is an *invert control*. This oligo has the advantage of possessing the same base sequence as the specific MO, but in reverse order, and therefore displays the same GC%, G content and self-complementarity, without binding to the target mRNA sequence. However it is important to perform a homology search to ensure it will not hold complementarity to unintended targets. Notice that *an invert sequence is not a sense,* which is actually not recommended as a negative control [117].

A *five-nucleotide mismatch* oligo is also widely used as a control. When designing this type of oligo, the five mismatched bases should be distributed relatively evenly throughout the sequence of the MO, as long stretches of complementarity could cause a partial downregulation of the target.

An additional approach is to use two different MOs that target the same mRNA. These can be two non-overlapping translation-blockers or one translation-blocker and one splice-blocker. If the same phenotype is observed with both MOs, this provides a good indication that the results are due to the downregulation of the specific protein. Moreover, the two MOs can also be delivered in combination, allowing the use of each one at a concentration lower than their effective dose. In principle, this should minimize the chances of mismatch for each while still achieving the desired specific effect.

It is currently accepted that a reasonable experimental design should include at least two negative controls and two non-overlapping specific MOs [123].

Finally, an ideal experiment would also include a rescue strategy. This could be achieved for example by co-electroporating a MO that binds a sequence within the 5’UTR, together with the specific mRNA that has been made resistant to it by deleting that region. However, if the phenotypic rescue is not total, the culprit could also be on problems other than unspecificity such as dosage, timing or uneven delivery. Alternatively, the link between phenotype and specific protein downregulation can be further supported by complementary evidence, for example by knocking down expression by different means.

### Analysis: tips and tricks

When analyzing the results of this type of loss-of-function experiment, it is important to consider that the amount of MO taken up by individual cells in a tissue may not be even. Moreover, MO incorporation (and potentially the extent of phenotypic effect) may vary from embryo to embryo. This variability can be due to many factors such as the volume and concentration of the injected solution, backflow from the neuropore or the injection site, placement of the electrodes, etc. [120]. Therefore, it is good practice to analyze a reasonably large number of embryos, and if possible consider only those in which adequate MO incorporation or efficacy can be verified.

Incorporation can be easily verified when using fluorescently tagged MOs by examining whole mounts or sections under the microscope (Figure 4E-H). However, the fluorescence signal is lost over time because of the dilution of the MO concentration inside the cells as a consequence of proliferation. Therefore, we are able to confidently detect it in developing chick eyes for only about 2 days after electroporation. At longer time points, the intracellular concentration of MO might still be high enough to elicit an effect, but not to be detected by these means. In our experience, this problem can sometimes be overcome by immunohistochemical signal amplification using an antibody against fluorescein [86]. Downregulation efficacy on the other hand can be evaluated by immunoblots and immunohistochemistry, as long as an antibody specific for the protein of interest is available. For splice-blocking MOs, an RT-PCR reaction can be designed to assess the effect of the treatment on the structure of the target mRNA (reviewed by [117]).

A further consideration is that of the directionality of migration of MOs during electroporation. Bearing in mind that, unlike nucleic acids, MOs are electrically neutral, the expectation would be that they get incorporated in the cytoplasm by diffusion as pores open in the plasma membrane. However, we and others have consistently observed a directional migration in the electric field [86,119,120,127]. Such directionality is an advantage, since it allows for better control when targeting a specific tissue. One possible explanation is that the light charge of the end modification might be enough to produce this effect. Another possibility is that since these molecules are so small, they are easily carried by the movement of fluids inside the embryo elicited by the electric field. It is important to mention that, during injection, part of the solution can escape the neural tube through the anterior neuropore and be incorporated by the surface ectoderm on the contralateral side.

### Alternatives for loss-of-function studies

Other commonly used strategies to downregulate expression of specific genes include the electroporation of dominant-negative constructs, antisense oligonucleotides and RNAi.

The dominant-negative approach is based on the idea of expressing a truncated or otherwise altered form of the protein under study, which will compete with the endogenous protein for its target or substrate without eliciting its normal effect (reviewed in [103]). This is not applicable in every situation and requires a good knowledge of the protein's structure and its functional domains.

Short sequence antisense oligonuclotides can bind to a target mRNA by base-pair complementarity and produce its degradation by endogenous RNAseH activity. DNA or RNA-based antisense strategies have reduced efficacy because of the rapid degradation of these molecules in the cytoplasm after administration. This problem can be overcome with the use of synthetic oligonucleotides, such as phosphorothioates, in which one of the nonbridging oxygens in the phosphate group is replaced by a sulfur, thereby increasing the stability of the polymer. On the other hand, RNA-mediated interference (or RNAi) takes advantage of the process by which short chains of double-stranded RNA (called siRNA), when delivered to a cell, can recruit the RNA-induced silencing complex (RISC) and bind by complementarity to a specific sequence in a target mRNA, eliciting its degradation or halting protein translation. This strategy has been very useful for studies of gene function in many animal models, including the chick [128]. However, both types of antisense technologies present the same type of weaknesses as MOs, with the additional drawbacks of lower stability, higher toxicity and in some cases higher risk of non-specific effects, and therefore careful sequence design and appropriate control experiments are essential for their successful application [129]. On the other hand, plasmid and viral vectors encoding short hairpin RNA (shRNA) can be used to overcome the problem of low stability, and in the case of retroviral vectors, achieve long-term expression of the silencing transgene ( [114,130-133]; and others). Moreover, this kind of approach allows the tracking of silenced cells as a marker gene can be linked to the shRNA expression cassette [130]. For detailed reviews and protocols on shRNA-based approaches, see references [99,103,113-115,130,134,135].

## Applications of the chick primary retinal cultures

*In vitro* culture systems constitute very powerful tools that do not replace but complement in vivo studies, in order to further our understanding of biological processes. Moreover, they have important additional applications, including drug development and the identification of factors that promote cell survival and differentiation.

The dissociation and *in vitro* culture of retinal cells from embryonic chick eyes provides an excellent system to study the mechanisms that regulate the survival, cell-fate determination and sequential differentiation of retinal progenitors in a cell-autonomous way, and with good control of the cellular microenvironment (reviewed in [136]). The nature of these cultures allows for a better discrimination between cell-intrinsic and extrinsic mechanisms, and facilitates electrophysiological recording in individual cells. Altogether, these characteristics make primary retinal cultures particularly attractive for high throughput screening purposes and applications related to drug discovery. What is more, this system has been successfully applied to the identification and characterization of several factors that enhance or favor specific developmental processes, such as the differentiation or survival of specific cell types. Examples of this include the identification of rod-derived cone viability factor (RdCVF) [137-139], as well as the characterization of other factors that can modulate retinal cell survival such as retinoids, lens epithelium-derived growth factor (LEDGF), adenosine, nitric oxide and components of the interphotoreceptor matrix [140-144], and modulators of photoreceptor cell differentiation such as ciliary neurotrophic factor (CNTF), neurogenin1 and certain homeobox transcription factors [145-147], among others.

The protocol for dissociated chick retinal cell culture was developed by Ruben Adler and colleagues in the 1980s, and further perfected by his and other groups over the following decades [148-150]. These cultures have been primarily characterized using eyes of ED5-8 embryos (H&H stage 27–34). The procedure consists of carefully removing the RPE, lens and vitreous from enucleated eyes, cutting the neural retina in small pieces and trypsinizing the tissue to obtain dissociated cells, which are then plated in polyornithine-coated tissue culture dishes to form low-density monolayers, and cultured in serum-supplemented or serum-free medium at 37°C (for a detailed protocol see Additional file 4). It is important to point out that the predominant photoreceptor types in chicks are cones, which constitute about 86% of all photoreceptors in this animal [151], and that the percentages of photoreceptors versus retinal neurons in these cultures varies with the age at dissection, with a higher percentage of cells differentiating as photoreceptors at earlier stages and this percentage decreasing over time (photoreceptors represent about 70-80% of all differentiated cells at ED5-6, and only about 30% of them at ED8) [152-154].

In order to take full advantage of the potential of this system to study gene function, the availability of efficient transfection techniques that permit the manipulation of gene expression becomes essential. Transfecting retinal cells in culture using the RCAS technology is not adequate, since they do not proliferate under these culture conditions and therefore cannot be infected by these viruses. On the other hand, electroporating dissociated retinal cells from chick and other animals is possible, though it is generally inefficient. Alternatively, calcium phosphate-mediated transfection can be employed in this system, but it has the disadvantage of high toxicity [155], whereas some lipid-mediated techniques perform better in that regard but have been reported to achieve efficiencies in the order of just 4% [156].

A different approach is to perform the viral infection or electroporation in the animal in vivo, as described in the previous sections, and then proceed with the culture. This is a useful strategy, but it is limited by the fact that transfection needs to be done at earlier stages, since it takes time for the viral infection to extend to a large portion of the cells, and electroporation is challenging and inefficient at stages later than ED4 because of the position of the embryo and the obstruction by embryonic membranes. This lag between transfection and culture implies that these methods are not the best choice to differentiate between primary and secondary effects of the regulation of the gene of interest, and to study phenomena such as the differentiation potential of precursor cells.

## Realizing the full potential of the chick embryo as a developmental model

The establishment of the gain- and loss-of-function techniques discussed above brought about the capacity to manipulate gene expression in the chick embryo in a manner that is rapid, efficient and cost effective. However, the biggest strength of this system resides in the possibility of combining these approaches with the well-established embryological manipulations and ex-ovo culture methods for which the chick is well known, and with the newly developed bioinformatics resources and genomic data sets that have become available in the recent years.

A variety of methods have been devised for the ex-ovo culture of whole chick embryos in order to improve their visualization and accessibility for experimental manipulation while maintaining the in vivo context. Some of the classic approaches are derived from the work of Denis New, and allow the culture of early stage embryos (starting from unincubated eggs) for up to 3 days (stage 15 to 17 on average). These cultures have been extensively used in developmental research, but they have the limitations of being useful only for studies focused on early developmental time points and of making only the ventral side of the epiblast directly accessible for manipulation [157-160]. Later modifications of this technique include early chick (EC) cultures, which are simpler and faster than New culture and provide the option of dorsal-side-up positioning of the embryo [161,162]. Other techniques have also been developed for use in older embryos, including culture in surrogate eggshells to improve hatchability of experimentally manipulated chicks [163] and shell-less cultures that use a glass bowl for culturing chicks of embryonic day 2 to 5 [164], or that use a polyurethane membrane affixed to a plastic cup as a vessel for culturing embryos for at least 14 days [165], among others. These methods can be used in conjunction with morpholino or plasmid electroporation [166,167], thus overcoming some of the restrictions of those techniques when applied in ovo, such as proper electrode positioning to target dorsal vs. ventral embryonic structures at very early stages, or the eye at later time points. Such strategies would provide a unique scenario for the study of molecular and cellular mechanisms regulating early stages of eye development, such as eye field specification, and optic vesicle/optic cup formation, or later aspects of retinal cell differentiation such as synaptogenesis, to a level of analysis that has been so far challenging to achieve in most animal models.

In addition, microsurgical manipulations such as tissue grafting, ablation, transplantation and chimeras have been well established for this animal model and are extremely informative in the study of developmental processes ( [6,168-176], and others). Such techniques can be performed in ovo, though ex-ovo cultures expand their application to cases in which the tissue of interest is not easily accessible at the desired stage. The combination of these methods with genetic manipulations raise interesting possibilities for experimental design that are particularly suitable for the study of tissue interactions, cell autonomy, position effects and signaling, and that are either not available or difficult to accomplish in other systems [177]. For example, Fekete and Cepko created intraspecific chimeras by transplanting restricted portions of donor chick embryos to hosts with a different susceptibility to RCAS virus infection [57]. In this way, when infection was attempted either before or after transplantation, only the tissues derived from the susceptible donor strain expressed the transgene, providing a paradigm that can be used in fate mapping, cell tracking or when transgene expression needs to be restricted to certain tissues.

One aspect in which the chick model can offer considerable advantages is in the rapid analysis of promoters and enhancers [98,106-108]. In silico comparison of genomic sequences among different species is frequently used to predict cis-regulatory elements, which are recognized as highly conserved, non-coding DNA sequences [107,108,178,179]. The premise is that sequence blocks that are critical for regulation of important developmental genes can survive evolutionary pressures. In this sense, human/mouse comparisons are of great value, but the high sequence conservation between these species make it difficult to identify functional elements among these non-coding blocks. Therefore, genomic comparisons with species that are separated by a wider phylogenetic distance, such as Xenopus, Zebrafish, or chick, can be very valuable in pinpointing functionally relevant regulatory sequences [108,178,179]. In addition the chick, being an amniote, can be more instructive in the identification of elements that play important developmental roles within this group, and its compact genome facilitates the functional characterization of these elements.

An excellent example of the power of the chick model when taking full advantage of its versatility is the work of Uchikawa et al. on the analysis of enhancers for the gene Sox2 [180]. In this article the authors sequenced a 50-kb region of the chick genome covering the Sox2 locus and scanned it for enhancer activity by electroporation of reporter constructs carrying various genomic fragments of that locus. New culture was used for electroporation at early developmental stages, whereas *in ovo* electroporation was used to study enhancers that are active later in development. The expression pattern of the reporter gene was compared to the normal pattern of expression of Sox2 as assessed by in situ hybridization. In addition, a chick-quail transplantation system was used to confirm the induction of the activity of one of the identified enhancers by the Hensen's node. Finally, the nucleotide sequences of the Sox2-flanking region of mouse, human and chick were compared, verifying the high degree of conservation of the identified sequence blocks.

Recently, the sequencing of the chicken genome and the development of new technologies such as microarrays and high throughput DNA sequencing have broadened the potential of this animal model. It is now feasible to carry out functional studies on the role of a gene or regulatory sequence with extremely high spatiotemporal resolution, ease and speed by combining gain- and loss-of-function strategies with more traditional embryonic manipulations, and to pursue a more comprehensive level of phenotypical analysis including, though not limited to, *in vivo* live imaging followed by assessment of global changes at the transcriptome and/or epigenome level.

The potential of these approaches for the study of eye development has not yet been fully realized, but their application is likely to bring about significant progress in the field.

## Conclusions

The chick embryo has become one of the most versatile systems in developmental biology. This is due to its intrinsic characteristics as an animal model, and to the development of powerful techniques for gain- and loss-of-function of gene expression, both *in vivo* and *in vitro*. In this article, we have presented an overview of some of the technological advances that have been responsible for major contributions to the fields of developmental neurobiology and ophthalmology using the embryonic chick. Multiple options are available for the study of gene function in this model, and thus, the choice of research strategy depends on several factors including: (1) the biology of the system under investigation, i.e., the proliferative characteristics of the tissue, its degree of differentiation, accessibility for manipulation, etc.; (2) the timing of the treatment and the experimental time window (short vs. long term); (3) the type of question that needs to be answered. With the variety and flexibility of experimental methods available for the embryonic chick, it is clear that this ancient model system will continue to be at the center of developmental research for the years to come.

## Competing interests

The authors declare that they have no competing interests.

## Authors’ contributions

Both MNV and MVC-S contributed to the writing of the manuscript, preparation of figures and development of technical protocols. Both authors read and approved the final manuscript.

## Supplementary Material

Additional file 1 RT-PCR protocol for the detection of ALV viral particles. Click here for file

Additional file 2 Protocol for the preparation of live RCAS virus stocks. Click here for file

Additional file 3 Protocol for the determination of RCAS stock titer. Click here for file

Additional file 4 Protocol for the primary culture of chick retinal cells. Click here for file
