# Supplementary material for: Rediscovering the chick embryo as a model to study retinal development
Source: Neural Dev. 2012 Jun 27;7:22. doi: 10.1186/1749-8104-7-22 (PMC3541172; doi:10.1186/1749-8104-7-22)
Supplement: Additional file 1 — RT-PCR protocol for the detection of ALV viral particles. [file 1749-8104-7-22-S1.pdf]

RT-PCR protocol for the detection of ALV viral particles.

Collection of amniotic fluid samples

- Use ED6 eggs or older (in younger embryos the amniotic cavity is still very small making obtention of amniotic fluid more difficult)
- Thoroughly clean the egg shell surface with alcohol wipes.
- Open eggs through the air chamber and locate the amniotic cavity. Using a 1 ml syringe and a 26 gauge × 3/8" needle collect amniotic fluid.
- Centrifuge at 12,000 rpm for 15 min at 4°C to obtain amniotic fluid devoid of cellular debris.
- Collect supernatant and use immediately or store at -20°C.

NOTE: It is important to avoid any potential contamination of the samples with viral particles that may be present in the laboratory environment as well as possible cross-contamination between eggs. In order to ensure this, the egg shell should be thoroughly cleaned, forceps and scissors should be sterilized before each sample collection, and new sterile syringes and needles should be used for each embryo.

RT-PCR for ALV transcripts

- Take 20 μl of centrifuged amniotic fluid and process for DNA digestion with DNAseI (Invitrogen, Carlsbad, CA) according to manufacturer instructions.
- Divide the sample in two aliquots of 10 μl.
- Process one aliquot for reverse transcription with hexamer random primers and cloned AMV reverse transcriptase (Invitrogen, Carlsbad, CA) following manufacturer's instruction. The use of cloned AMV is recommended since in our experience, SuperScript II is less efficient than cloned AMV for this purpose, and native AMV leads to unspecific false positive amplification.
- Process the second aliquot in parallel without including cloned AMV reverse transcriptase to use as control for possible "false positives" from DNA amplification.
- Take 2 μl of reverse transcribed reaction (or reactions lacking reverse transcriptase) to use as template for PCR reactions.
- Primers and parameters for PCR amplification can be found in McNally et al 2010.
