# Supplementary material for: Rediscovering the chick embryo as a model to study retinal development
Source: Neural Dev. 2012 Jun 27;7:22. doi: 10.1186/1749-8104-7-22 (PMC3541172; doi:10.1186/1749-8104-7-22)
Supplement: Additional file 2 — Protocol for the preparation of live RCAS virus stocks. [file 1749-8104-7-22-S2.pdf]

Protocol for the preparation of live RCAS virus stocks.

- Culture DF-1 cells according to protocols provided by ATCC.
- Seed cells in 60mm dishes until they reach 80-90% confluency and transfect with RCAS plasmid using Lipofectamine 2000 according to manufacturer's instructions.
- The next day, split cells from one 60mm dish into two 100mm dishes to grow the virus. Alternatively, half the volume from a 60 mm dish can be seeded into one 100 mm dish and the other half into 3-4 35mm dishes to test viral infection and expression of the inserted gene by immunohystochemistry and Western blot.
- Incubate cultures for 1 or 2 days, until they reach 80-90% confluency. Don't let cultures overgrow to avoid the release of possible toxic molecules from dead cells.
- Collect supernatant into Amicon ULTRA-15 (100K) filters (Millipore). Add 15ml (max volume) to each tube and spin at 3000g for 30min at 4°C. Recovery volume is usually approximately 200ul.
- Divide in 20µl aliquots and store at -80°C.

NOTE: After collecting the supernatant, the cells can be split again for a second virus harvest. Once obtained, the viral stocks should be divided into single use aliquots and kept frozen at -80° C. Repeated freeze-thaw cycles should be avoided, and while in use the working aliquot should be kept on ice to avoid loss of viral activity. It is not advised to keep viral stocks for more than a day or two at 4°C.
