# Supplementary material for: Rediscovering the chick embryo as a model to study retinal development
Source: Neural Dev. 2012 Jun 27;7:22. doi: 10.1186/1749-8104-7-22 (PMC3541172; doi:10.1186/1749-8104-7-22)
Supplement: Additional file 3 — Protocol for the determination of RCAS stock titer. [file 1749-8104-7-22-S3.pdf]

Protocol for the determination of RCAS stock titer.

Day1

- Seed DF-1 cells in a 24 well plate at a 25% confluency using 1ml of medium (90% DMEM with 10% heat inactivated fetal calf serum) per well.
- Make sure to prepare enough wells to be able to test at least 6 different dilutions of virus in duplicate, plus a couple of extra wells for the negative controls including: i) no virus infection processed for immunohistochemistry with primary and secondary antibodies; ii) infection with a high viral concentration and processed only with the secondary antibody.

Day2

- Make serial dilutions of the viral stock in medium (a dilution range from 10^{-5} to 10^{-10} is usually appropriate). Prepare at least 2 ml of each viral dilution (enough to perform the experiment at least in duplicate).
- Replace the medium in the wells with 1ml per well of the diluted virus solution.
- Incubate for 2 days as recommended for DF-1 cells.

Day 4

- Remove the medium, rinse the cells once in warm Hank's Buffered Salt Solution (HBSS), remove buffer and fix the cells for 10 min in 4% paraformaldehyde. Then wash several times in PBS.
- Process for immunohistochemistry against viral proteins, such as p19 or p27 and stain with DAPI.

Titer calculation

- Examine under fluorescence microscope and determine the highest dilution in which positive cells can still be observed. The titer is expressed as the inverse of that dilution. Example: If the last dilution in which positive cells are observed is 10^{-7}, and the volume of medium in the well is 1 ml, then the viral titer will be 10^{7} infective particles/ml.
