# Supplementary material for: Rediscovering the chick embryo as a model to study retinal development
Source: Neural Dev. 2012 Jun 27;7:22. doi: 10.1186/1749-8104-7-22 (PMC3541172; doi:10.1186/1749-8104-7-22)
Supplement: Additional file 4 — Protocol for the primary culture of chick retinal cells. [file 1749-8104-7-22-S4.pdf]

Protocol for the primary culture of chick retinal cells.

Media and solutions

- Pen/Glu: Mix 1.8g NaCl, 3.0g L-Glutamine (15mg/ml OR 100mM; Fisher Scientific), and 0.631g Penicillin-G (3.15mg/ml; Invitrogen). Add ddH20 to 200ml. Mix and filter sterilize. Aliquot and store at -20°C until needed.
- Medium 199 Pen/Glu: To 250 ml M199 (Sigma) add 5.8 ml of Pen/Glu.
- 2XPhR medium (100ml): To 90ml M199 Pen/Glu add 10 ml Fetal Calf Serum and 900 ul linoleic acid-albumin (9ul/ml, Sigma).
- 20X Polyornithine stock solution (l mg/ml; poly-L-ornithine in boric acid buffer): Add 0.2g poly-L-ornithine to 200ml of 0.15 M boric acid buffer pH 8.4. Filter sterilize. Aliquot and refrigerate up to a year.

Preparation of TC dishes

- Add 2ml of 1X Polyornithine solution (diluted from 20X stock with sterile water) to each tissue culture (TC) quality 35 mm dish, and incubate for 3 hours at room temperature.
- Wash with sterile H_2O (at this point dishes can be stored in 4°*C* for extended periods of time).
- Add 2ml Medium 199 Pen/Glu and place in incubator overnight.
- Remove media and add 1ml of 2X PhR media prior to dissection. Keep in incubator until ready to seed cells.

Retinal dissociation and culture

- Enucleate eyes in 1X HBSS or CMF (Calcium-Magnesium-free HBSS; CMF helps the RPE detach from the retina) .
- Transfer retina to 1X CMF and cut into small pieces with tungsten needles.
- Using a siliconized glass Pasteur pipette, transfer retina pieces to a 15ml tube with CMF and incubate in water bath for 10 minutes.
- Take the tube out of the water bath and remove the solution without disturbing the tissue at the bottom of the tube.
- Add Dnase/Trypsin solution to the tube, without disturbing the tissue pellet; incubate 20 minutes at 37°C.
- During incubation:
  - flame bores of three siliconized, plugged pipets to reduce bore size.
  - Fill 50ml conical tube with Medium 199 with Pen/Glu: 5 ml for each ED8 eye, 2 ml for each ED6 eye.
- Add about 5 ml of LEBM- 1%BSA (without removing Trypsin/DnaseI) to tube.
- Remove all medium, being careful not to disturb the tissue. Add 5 more ml of LEBM-1%BSA and remove again. Add another 2 ml and then remove most of the medium.
- Take a smaller bore pipette and fill with LEBM- 1%BSA.
- Insert pipette to the bottom of the tube and dissociate by gently pipetting up and down 20 times, being careful not to form air bubbles.
- Fill a 50ml conical tube with aproximately 30 ml medium 199 Pen/Glu. Place a Falcon cell strainer on the top of the tubeand wet with some medium 199.
- Use a glass pipette to gently pass cell solution through filter. Add more Medium 199 until the 50ml tube contains about 40ml.

- Take out a small aliquot of the cell solution and fill hemocytometer to count cells.
- Plate approximately 600,000 cells into each polyornithine-coated 35 mm tissue culture dish (low density culture).
